# Fidgetin-Like1 Is a Strong Candidate for a Dynamic Impairment of Male Meiosis Leading to Reduced Testis Weight in Mice

**DOI:** 10.1371/journal.pone.0027582

**Published:** 2011-11-16

**Authors:** David L'Hôte, Magalie Vatin, Jana Auer, Johan Castille, Bruno Passet, Xavier Montagutelli, Catherine Serres, Daniel Vaiman

**Affiliations:** 1 CNRS UMR 7592, Institut Jacques Monod, Equipe Génétique et Génomique du Développement Gonadique, Paris, France; 2 Université Paris Diderot-Paris VII, Paris, France; 3 U1016 Département de Génétique et Développement, Institut Cochin, INSERM, Paris, France; 4 Université Paris Descartes, Paris, France; 5 Unité de Génétique des Mammifères, Institut Pasteur, Paris, France; 6 Département de Génétique des Animaux, INRA, Jouy-en-Josas, France; Cornell University College of Veterinary Medicine, United States of America

## Abstract

**Background:**

In a previous work, using an interspecific recombinant congenic mouse model, we reported a genomic region of 23 Mb on mouse chromosome 11 implicated in testis weight decrease and moderate teratozoospermia (∼20–30%), a Quantitative Trait Locus (QTL) called Ltw1. The objective of the present study is to identify the gene underlying this phenotype.

**Results:**

In the present study, we refined the QTL position to a 5 Mb fragment encompassing only 11 genes. We showed that the low testis weight phenotype was due to kinetic alterations occurring during the first wave of the spermatogenesis where we could point out to an abnormal lengthening of spermatocyte prophase. We identify Fidgetin-like 1 (Fignl1) as the gene underlying the phenotype, since if fulfilled both the physiological and molecular characteristics required. Indeed, amongst the 11 positional candidates it is the only gene that is expressed during meiosis at the spermatocyte stage, and that presents with non-synonymous coding variations differentiating the two mouse strains at the origin of the cross.

**Conclusions:**

This work prompted us to propose Fignl1 as a novel actor in mammal's male meiosis dynamics which has fundamental interest. Besides, this gene is a new potential candidate for human infertilities caused by teratozoospermia and blockades of spermatogenesis. In addition this study demonstrates that interspecific models may be useful for understanding complex quantitative traits.

## Introduction

Fertility is an important concern both in human medicine, where 10–15% of the couples call for the services of assisted reproductive technologies, and for various domestic species of economic interest, such as dairy cows where a striking drop in fertility has been observed in recent years [1], [2]. In humans, the genetic basis of male infertilities is far from being completely elucidated and is mostly explained by deletions of the AZF region of the Y chromosome [3], [4]. However, such infertilities affect no more than 10% of the male patients, stressing the need of identifying new actors responsible for these disorders. Hundreds of genes involved in gametogenesis in a broad sense have been identified by gene-invalidation approaches in mice [5]. But, while these experiments lead to a complete abolishment of gene expression, more quantitative approaches are of interest to identify new fertility genetic determinants. Up to now, several loci implicated in the quantitative regulation of both male and female fertility traits have been mapped in the mouse genome, thanks to crosses between closely related strains. However the genetic bases underlying these QTL (Quantitative Trait Loci) have generally not been discovered so far [6].

Different types of mouse models have been developed for accelerating positional cloning procedures, and are well-suited for identifying gene(s) governing quantitatively fertility traits. Amongst the common mapping tools existing in plant and animals, panels of inter-specific recombinant congenic strains (IRCS) have been developed. Such panels thanks to their interspecific origin, exhibit a much higher genetic diversity than intra-specific panels, thus enhancing the contrasts between phenotypes [7]. This is the case of a panel of 53 recombinant congenic mouse strains harboring a small quantity of *Mus spretus* genomic fragments dispersed in a *Mus musculus domesticus* background. This panel of IRCS has been developed at the Pasteur Institute (Paris, France) from an original cross between the SEG/Pas strain (SEG) of *Mus spretus* origin and the C57BL6/J (B6) *Mus musculus* strain [7], [8]. On average, each IRCS genome is composed of ∼1.5% of *Mus spretus* genome, distributed in 1 to 8 fragments in a C57B6/J context. *Mus spretus* and *Mus musculus* diverged about 2 million years ago, this divergence leading to the existence of a nucleotide substitution roughly every 80 bp, close to that existing between humans and chimps [9]. This important diversity and the weak proportion of *Mus spretus* genome dispersed in B6 background make it possible to rapidly map QTL in the centimorgan range [10].

In a previous study, we reported on the mapping of a low testis weight QTL (*Ltw1*) mapping on mouse chromosome 11 (between 3.7 Mb and 26.5 Mb) following the phenotypic analysis of the 97 C strain [10]. This strain exhibits a significant reduction in absolute and relative testis weight compared to the B6 control, associated with a reduction in the seminiferous tubules' diameter. We then strived to identify the gene(s) responsible for this low testicular weight phenotype. For this, a double approach was undertaken aiming (1) at refining the location of the critical region by creation of recombinant events inside the *spretus* fragment of the 97 C mice, in parallel with (2) a thorough phenotypic analysis of the testis in order to pinpoint and characterize as precisely as possible the features differentiating 97 C and B6 mice. A combination of these cartographic and phenotypic approaches, together with a careful exclusion of most the genes present in the minimal remaining fragment, called attention to the *Fidgetin-like1* gene (*fignl1*) as the best candidate for explaining the 97 C testis phenotype. Here, we propose that *Fidgetin-like1* polymorphisms differentiating the two mouse strains are responsible for the meiosis phenotype observed. In this study, we present arguments suggesting that the role of this gene is to control male meiosis dynamic.

## Results and Discussion

### 1) Fine phenotyping of 97 C testes reveals an impaired progression of the germ cells through the first wave of spermatogenesis

**Figure 1:**
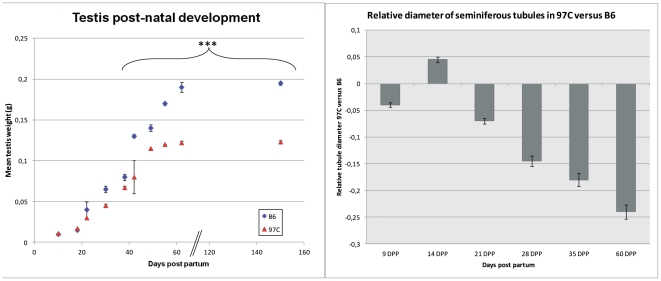
Testis development analysis of 97 C mice. The testis weight and the seminiferous tubule diameter were quantified in function of the age in B6 and 97 C IRCS mice. (a) The testis weight of B6 (▴) and 97 C (⧫) mice was expressed as a mean ± SEM of 3 to 4 animals per strain. *** indicates a significant difference with p<0.001 between 97 C and B6 mice. (b) Tubule diameters of B6 and 97 C mice were measured on 3–4 testes per strain on an average of 40 tubules. The results represent the ratio of the difference between 97 C and B6 mean values relative to B6 value. From 21 days post partum, the difference is significant (p<0.001).

We extended our preliminary observations of the 97 C phenotype [10] on an increased number of animals (n = 19) for testis histology and seminal vesicle secretion. This analysis did not reveal any obvious abnormality concerning spermatogenesis in 97 C mice and confirmed the observation of a clear differences in testis weight between 97 C and B6 adult mice (0.12 g instead of 0.18 g, corresponding to a difference of ∼30%, Figure 1a). Animals harbored a normal epididymal sperm reserve, associated to a teratozoospermia about 3 to 4 fold more elevated than in the B6 parent (20% vs 6%). Despite this recurrent defect affecting sperm head shape, 97 C animals were fertile, probably due to the high percentage of normal spermatozoa remaining in this strain. The sexual behavior of 97 C males was not different from the B6 parent, indeed, vaginal plugs were observed in mating experiment with B6 female, and 97 C male sired normal sized litters.

**Figure 2:**
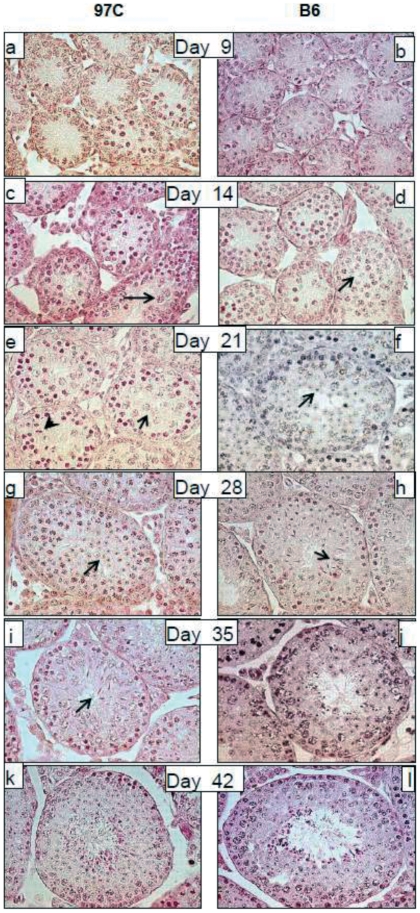
Impaired progression in the first wave of spermatogenesis in juvenile 97 C and B6 parental mice. A representative picture of seminiferous tubule histology of testes from 97 C (a,c,e,g,i,k) and B6 (b,d,f,h,j,l) mice at post natal day 9, 14, 21, 28, 35 and 42 is shown (objective ×60). At day 9 and 14, no difference was found between B6 and 97 C tubule cell population. A germ cell progression up to pachytene spermatocytes (arrows) was observable in both 97 C (c) and B6 (d) testes. At day 21, meiosis was completed and round spermatids (arrow in f) appeared in the tubule of B6, while spermatogenesis of 97 C was still in the meiotic phase with mostly germ cells at pachytene spermatocyte (arrow in e) or metaphase stage (arrow head in e). In addition, at this age, the lumen of the tubules was visible only in B6 mice. At day 28, spermatogenesis normally progressed in B6 up to the elongated spermatid stage (arrows in h) but displayed a one week delay in 97 C testes, where round spermatids (arrow in g) were the most advanced stage of germ cells present in the tubules. Elongated spermatids were not observed before the fifth week in post-natal testis of 97 C (i). Figures of spermiation seen at day 35 in control testes (h) signed the end of the first wave of spermatogenesis in B6 mice, which was only attained at the sixth week in 97 C mice (k). From this time, testis histology of the two strain was comparable (k and l) but a smaller tubule diameter was noticeable for the 97 C testis.

**Figure 3:**
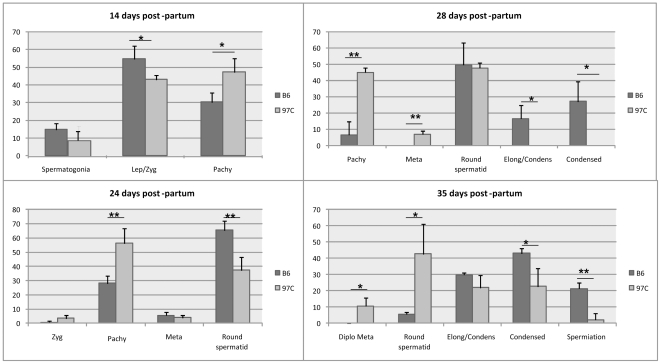
Distribution of the different stages of the spermatogenesis during the establishment of the first wave from 14 days post partum to 35 days post partum in testes of B6 (black bars) and 97 C (grey bars) mice. The results represent mean ± SD of the frequency of each stages observed in the testes of 3 or 4 males for each strain (with 50 to 130 tubule sections observed by male at each age). Abbreviation: Lep = leptotene; Zyg = zygotene; Pachy = pachytene; Diplo = diplotene; Meta = metaphase; Round spermatid corresponds to the steps 1 to 7 of the spermiogenesis; Elong/condens = elongating and condensing spermatid of steps 8 to 12 of the spermiogenesis; Condensed = condensed spermatid of steps 13 to16 of spermiogenesis. Different between B6 and 97 C at:* p<0.05; ** p<0.01; *** p<0.001 with student t test.

Since our previous characterization of the 97 C testicular phenotype was limited to adult mice [10], we examined the postnatal testis development in 97 C mice in order to detect putative early defects at the origin of the adult phenotype. Testes were analyzed at various time points from birth until two months (Figure 1). The testis weight evolution in 97 C mice was apparently normal from 9 to 16 days post partum (DPP) but was delayed from 21 DPP. This difference became statistically significant from 28 DPP and remained significant in older mice. 97 C testis weight reached a plateau at 6 weeks, exhibiting a value about 30% lower than B6's in adult mice (Figure 1a). In parallel with testis weight, the mean diameter of seminiferous tubules in 97 C mice was significantly smaller than this of B6 from 21 DPP (∼7%) and this difference reached 25–30% thereafter (Figure 1b). Since this time period corresponds to the onset of the first wave of spermatogenesis, we examined in detail histological sections during this period (figure 2) and we quantified the seminiferous tubule sections according to the stage of the spermatogenesis cycle (figure 3). No obvious difference was observed between 97 C and the B6 testis histology at 9 DPP and 14 DPP (figure 2 a to d). At 14 DPP we observed tubules containing spermatocytes in similar frequency in 97 C and B6 testes (90.5±4.61% vs 85.0±3.46%, respectively), at leptotene, zygotene and pachytene stages (figure 3a) suggesting that the proliferative phase of spermatogenesis and the entry in meiosis occur normally. The first sign of dysfunction in 97 C spermatogenesis was detected after the third week after birth. At 24 DPP, we observed in B6 testes a majority of sections with round spermatids (figure 2f and figure 3b) whereas in 97 C testes, the tubules containing this post meiotic stage were significantly less frequent (65.6±6.56% vs 37.37±9.08% respectively, P<0.001 (figure 3b)). We could still detect in 97 C testes a majority of tubules with spermatocytes as the most advanced stage, notably early (first mid) pachytene stage, which were less frequent in B6 testes (56.2±10.35% vs 28.07±5.30% respectively, P<0.001). At 28 DPP, tubule sections of 97 C testes containing spermatocytes were still observed, at the same frequency than those with round spermatids (45.1±2.69% vs 47.7±3.11% respectively) (figure 2g and figure 3c). At this same period in B6 testes, spermiogenesis concerned more than 90% of the germ cells and was already advanced to the elongated and condensated spermatid stage (13–15 step) (figure 2h and figure 3c). At 35 DPP, half of the observed sections of 97 C testis still contained both delayed meiotic cells and round spermatids (step 1–7), stages nearly absent in B6 testis (figure 3d) The release of testicular spermatozoa in the tubule lumen was visible at 35 DPP for B6 and 42 DPP for 97 C (figure 2j and h). Consistently with this time-lag in spermatogenesis differentiating the two strains, the central lumen of tubules in 97 C testes was not formed before 4 weeks, whereas in B6 testis the lumen was opened from the third week.

The examination of epididymis sections over the same period of time (2 to 6 weeks) highlights a one week delay in the apparition of spermatozoa in the epididymal duct of 97 C compared to B6 mice (week 6 versus week 5, respectively) (Figure S1).

Examination of seminiferous tubule sections of SEG strain at 14, 24 and 28 DPP did not reveal difference in the time course of spermatogenesis compared with B6 mice (data not shown).

**Figure 4:**
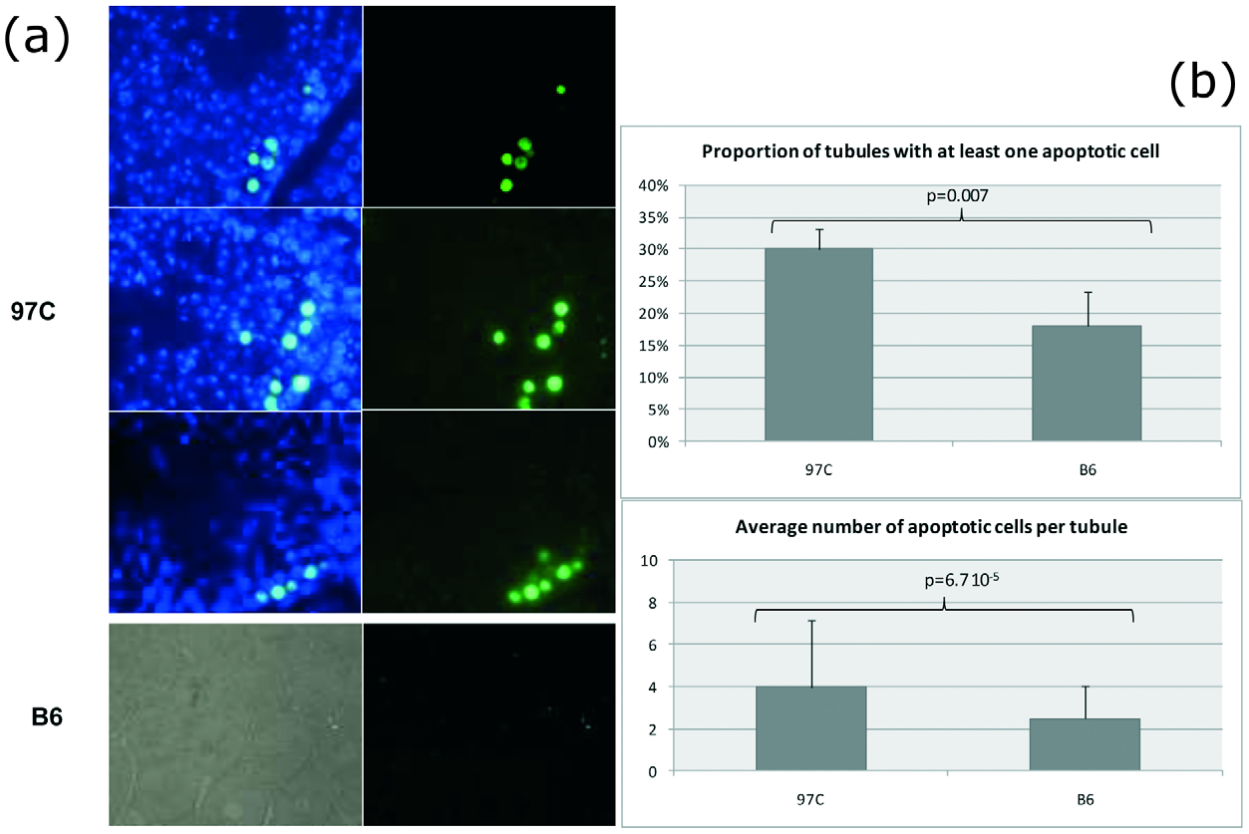
Apoptosis in the seminiferous tubules of adult 97 C mice. The apoptotic cells were studied for their localization (a) and their number (b) in B6 and 97 C testes using the TUNEL assay. (**a**) DNA strand breaks of the apoptotic cells were revealed by fluorescein-dUTP (green fluorescence) and the sections were counter-colored by DAPI (blue fluorescence). Clusters of TUNEL positive cells were observed in tubule sections of 97 C testes, principally spermatocytes at the stages VI–VII of the spermatogenesis (objective ×100). In B6 testis sections, the apoptotic cells were scattered in some tubules (left: transmission light; right: epifluorescence; objective ×20). (**b**) Percentage of sections containing at least one apoptotic cell was significantly higher in 97 C testis when compared to B6 (30% and 18% respectively; chi square test p<0.007). In these positive tubules, the number of apoptotic cells per tubule (mean ± SD) was significantly higher in 97 C testis than in B6's (3.98±3.15 and 2.48±1.54, respectively; p<6.7 10^−5^).

To determine whether the delay in the spermatogenesis development of 97 C mice was accompanied with apoptosis, we performed a TUNEL assay on adult testes (in post-natal rodent testis, cell apoptosis naturally occurs in spermatogonia and spermatocytes during the first wave of spermatogenesis, consequently we decided to assess apoptosis during adulthood in mature testis). The frequency of positive tubules (e.g. section where at least one apoptotic cell was detected) was significantly higher in 97 C testis when compared to B6 (0.30 and 0.18 respectively; chi square test P<0.007). Considering only positive sections, the mean number of apoptotic cells per tubule was significantly higher in 97 C testis than in B6's (3.98±3.15 and 2.48±1.54, respectively, P<6.7 10^−5^). This averaged rate masked a variable repartition among tubules, with apoptotic cells often observed in clusters in 97 C sections whereas they were rather scattered in B6 sections (Figure 4). The apoptotic cells in 97 C testes were mainly spermatocytes at mid-pachytene stage (see Figure 4). This increase in apoptotic cell number in 97 C, although clearly reflecting a dysfunction, is not sufficient to significantly reduce the sperm production of 97 C adult mice [10].

Taken together, these results show that in the 97 C testis, spermatogenesis progresses normally up to the pachytene spermatocyte stage, but a functional impairment in the first half of pachytene stages (still visible at 3 and 4 week) slow down the passage to late pachytene resulting in a lengthening of the meiosis duration. This defect, detected during the first wave of spermatogenesis becomes less evident in adult testis where successive spermatogenetic waves are overlapping. However the rate of apoptotic spermatocytes higher in adult 97 C testis than in B6'one attests for such a dysfunction. Thus, this early defect is never overcome and is presumably at the origin of the adult phenotype since the first wave of spermatogenesis and testicular growth are two correlated events. Such an association between meiosis delay and reduced adult testis weight has been observed, for example, in mouse mutants for TR4 genes [11]. In sum two possible effects could lead to the reduced testis weight: a slowing down of the passage from mitosis to meiosis of the spermatogonies, or a slowing down of the meiosis possibly inducing the observed increased apoptosis of germ cells in the seminiferous tubules, a hypothesis that we privilege to explain the phenotype.

As previously mentioned [10], 97 C males presented also with a seminal vesicle phenotype showing a translucent appearance instead of the normal off-white color visible in B6. The secretion of theses seminal vesicles was further examined by a 1D protein gel stained by Coomassie blue. No obvious difference was visible between B6 and 97 C.

### 2) Fine mapping of the low testis weight QTL (Ltw1) of 97 C line

**Figure 5:**
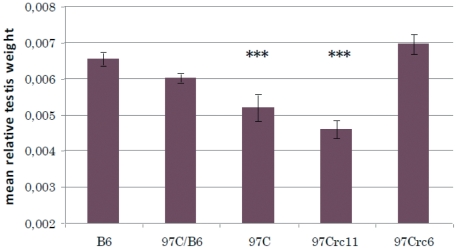
Relative Testis weight of parental B6 and IRCS 97Crc6, 97Crc11, 97 C. 97 C encompasses two chromosome segments of spretus origin on chromosomes 6 and 11. The 97Crc6 and 97Crc11 sub-recombinant were generated to separate the two fragments. Clearly the small testis weight phenotype segregates with chromosome 11. Results are expressed as mean ± SEM. Asteriks (***) denote strains that showed a significant difference when compared to B6 (P<5.10^−7^).

In a previous study, we have mapped a QTL of low testis weight (*Lwt1*) on a 23 Mb region of chromosome 11, thanks to the 97 C line supposed to harbor a unique *spretus* fragment on MMU11. The localization of this QTL was confirmed by the analysis of a F2 population [10]. On the occasion of an expressional study of testis genes of 97 C, we discovered an additional *spretus* fragment of approximately 9 Mb on MMU6 in this same strain, this fragment having been detected by its specific expression profile, compared to the B6 background [12]. In order to unambiguously map the *Ltw1* QTL, we created two congenic sub-strains harboring one *spretus* fragment, either on MMU6 or on MMU11 (called 97Crc6 and 97Crc11 respectively). Phenotypic analysis of these new strains showed that 97Crc6 mice exhibited only the seminal vesicle phenotype described above (abnormal liquid coloration) and that their mean testis weight was not significantly different from B6 (figure 5). As expected, the low testis weight phenotype segregated with the MMU11 *spretus* fragment harbored by the 97Crc11 strain (figure 5). We confirmed that 97Crc11mice exhibited the same abnormality of spermatogenesis that 97 C mice.

**Figure 6:**
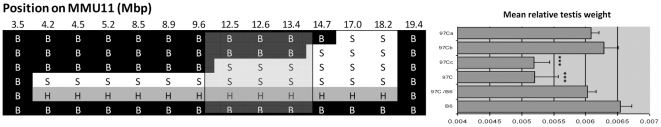
Fine mapping of the low testis weight QTL in inter-recombinant strains generated for this study from 97Crc11. Left panel: genotypes of the different recombinant lines (97Crc11a, b, c, noted 97Ca, b and c in the figure), parent B6, 97 C and heterozygote 97 C/B6. Black regions correspond to B6 background, white regions to homozygous *spretus* fragments and grey regions to heterozygous *spretus* fragments on chromosome 11. Marker positions are given in megabase pairs. Right panel: Histogram of the testis weight relative to body weight expressed as mean ± SEM for a minimum of 5 animals. *** denotes a significant difference when compared to B6 (p<10^−3^). By analyzing the fragment/phenotype segregation, it could be concluded that since 97 C and 97Crc11c exhibit the phenotype of low testis weight, the *Ltw1* QTL is located in a fragment of 5 Mb or less on MMU11 (hachured region between 9.6 Mb to 14.7 Mb).

In order to identify the genetic determinant of the *Ltw1* QTL, we first refined the MMU11 *spretus* fragment spreading over 23 Mb (3,539797 to 26,493225 bp) to a smaller QTL region of 16 Mb (3,539797 to 19,470000 bp) by genotyping microsatellites at the fragment boundaries. This region still encompasses 115 genes (either annotated or expressed sequences). For improving the mapping resolution, we backcrossed 97Crc11 mice with B6 parents in order to generate recombination events inside the MMU11 *spretus* DNA segment and then we derived from them homozygous sub-congenic strains. Three informative recombinant strains were generated at the homozygous state, 97Crc11a (14.7 Mbp–19.5 Mbp), 97Crc11b (13.4 Mbp–19.5 Mbp), 97Crc11c (9.6 Mbp–19.5 Mbp) (figure 6) and analyzed for the testicular phenotype. Only 97Crc11c exhibited a significantly smaller testis weight than that of B6 (figure 6) with a high level of teratozoospermia (35% of abnormal spermatozoa in average). The overlapping of the *spretus* segments of the 3 recombinant lines narrowed down the minimal interval of the *Ltw1* QTL to a 5 Mb *spretus* region located between microsatellites D11MIT259 and D11MIT148 (9.6–14.7 Mb). The reduced *Ltw1* QTL region of interest contains only 11 annotated genes according to the NCBI database (Table 1).

**Table 1:** List of genes and annoted sequences localized between 9.6 and 14.7 Mbp on Mouse Chromosome 11.

Gene symbol	Expression level in B6 testis (arbitrary fluorescence units)	Ratio of expression 97C/B6	Gene name
Vwc2	129	1.75	von Willebrand factor C domain containing 2
Zpbp	7259	0.77	zona pellucida binding protein
LOC432534	61	0.49	
Pms1Rik	10969	0.87	
4930512M02Rik	94	0.36	
Ikzf1 (Zfpn1a1)	137	0.83	IKAROS family zinc finger 1
Fignl1	16618	0.99	Fidgetin-like1
Ddc	75	0.83	Dopa decarboxylase
Grb10	1407	0.79	growth factor receptor-bound protein 10
Cobl	192	1.61	cordon-bleu
LOC63443	134	0.57	

### 3) Fidgetin-like1 is a pertinent positional and functional candidate gene susceptible to affect spermatogenesis in 97 C mice

To point out a pertinent candidate gene we choose to apply several screens considering expressional and functional data provided by experimental results, databases, and literature reports.

In a previous study, we performed a whole genome expressional analysis on B6 and 97 C testis [12]. We took profit of these data to quantify the expressional level of the 11 genes presents in the 5 Mb QTL region in B6 and 97 C testes (Table 1). Considering only transcripts with a fluorescence level >200 (arbitrary threshold for significant expression) on the Nimblegen arrays used, we noted that four genes *Vwc2*, *LOC634436*, *Ikzf1* and *Cobl* are expressed at a low or undetectable level in the B6 testis whereas four other genes, *Grb10*, *Zpbp*, *4930415F15Rik* and *Fignl1* are expressed at a high level (1,400 to 16,600 fluorescence unit, average fluorescence in the array ∼2300). For the rest of the study and taking into account its expression profile, we decided to call *4930415F15Rik* ‘Pms1’, for Post-Meiotic Spermatogenesis 1). These experimental results were consistent with the bioinformatic data (SymAtlas and Germonline data bases) reporting either no expression (for *Pms1, Vwc2, Ddc, Cobl, Ikzf1*) or a moderate (for *Grb10*) to strong (for *Fignl1*, *Zpbp* and *Pms1*) expression in the testis compared to other tested tissues.

**Figure 7:**
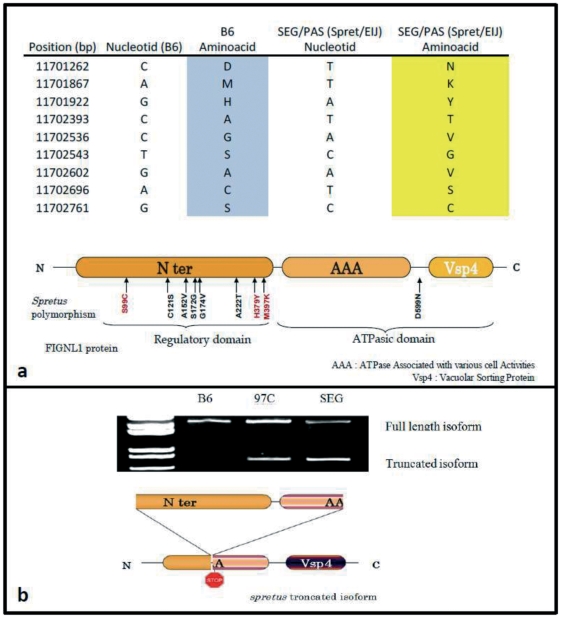
Specific features of spretus Fignl1. **a**) Polymorphism map of *spretus fignl1* gene. By PCR and sequencing of spretus Fignl1, we could observe 9 coding variants compared to the B6 version. These variants are mainly located (8 of them) in the N terminal region of Fignl1, reported as less conserved. In red are represented variants that could give rise to strong and significant variations in the protein structure or to post-translational modifications. S99C may generate disulphide bonds with other cystein residues, H379Y generates a phosphorylable tyrosine, and M397K is specifically encountered in Mus spretus, by contrast with all other species, including monotremes, birds, amphibians and fish (Figure S3). **b**) RT-PCR amplification of fignl1 ORF on testis cDNA of B6, SEG and 97C males, with primers spanning the complete ORF. The specific amplification product observed when a spretus allele is present corresponds to a mRNA smaller of about 1400 bp which may generate a truncated isoform of the protein; It is interesting to notice, that this isoform should not be problematic in spretus since it is normally present in this species.

The expression level of the four genes significantly expressed in the testis (*Grb10*, *Fignl1*, *Zpbp* and *Pms1*) was not different between 97 C and B6 mice as shown by the expression ratio 97 C/B6 which ranged from 0.8 to 1 (Table 1). Given this absence of deregulation, we attempted to identify sequence polymorphisms differentiating the *spretus* alleles from the *musculus* alleles of these genes. Systematic sequencing indicated that *spretus* alleles of *Zpbp* and *Grb10*, in SEG/Pas and 97 C strains, present only with synonymous variations by comparison with *musculus* alleles present in the B6 strain. By contrast, non synonymous polymorphisms were found in the two other genes, with one variation in *Pms1* (p. glu100val) and 9 differences in *Fignl1* (figure 7a). Moreover, we found that 97 C and SEG testes expressed an additional truncated isoform of *fignl1* corresponding to alternative splicing of Fignl1 mRNA, as revealed by sequencing (figure 7b).

In our search of the candidate gene, we could reasonably exclude *Vwc2*, *LOC634436*, *Ikzf1*, *Cobl*, *Zpbp* and *Grb10* genes estimating that their implication in the phenotype was improbable, due to their low level of expression in the testis *(Vwc2*, *LOC634436*, *Ikzf1* and *Cobl)*, or their absence of expressional deregulation in 97 C testis as well as their absence of non-synonymous polymorphisms (*Zpbp* and *Grb10*). This exclusion was strengthened by the results of gene invalidations for *Zpbp*, *Grb10* and *Ikzf1* which give rise to phenotypes far from that of 97 C mice. Indeed the knock-out of *Zbpb*, a gene principally expressed in post-meiotic germ cells, induces an aberrant acrosome biogenesis and dysmorphic spermatozoa, dissimilar to the characteristics observed in 97 C [13]. The disruption of *Grb10*, which encodes a growth factor receptor-binding protein, leads to a disproportionate overgrowth of some organs (placenta, muscle and pancreas) whereas the overexpression of some isoforms of the encoded protein results in growth suppression [14] a phenotype that was not observed for 97 C mice. At last, the mouse mutant for *Ikzf1* gene displayed an abnormality in the blood lineage with stem cells differentiating exclusively into erythroid and myeloid cells without providing lymphocyte cells [15], [16].

Thus, only two relevant potential candidate genes remained: *Pms1* and *Fignl1*. *Pms1* which is highly and specifically expressed in the mouse testis, presented with a post-meiotic expression at the spermatid stage. Indeed, during the first wave of spermatogenesis analyzed by RT-PCR, *Pms1* was undetectable until 20 DPP, a period where seminiferous tubules were principally populated by spermatogonies and spermatocytes and became detectable only afterwards (22–30 DPP) when the spermatids are present in addition to the precedent cells (Figure S2). We confirmed this expression pattern at the protein level by western blots using an antibody designed and characterized for the present study (Eurogentec ™) (data not shown). Therefore it is highly unlikely that a gene strictly expressed at a post-meiotic spermatid stage could affect a meiotic process. During this experimental work, we showed that the LOC432534 annotated gene is in fact a part of a *Pms1* transcript. Indeed we succeeded in amplifying by RT-PCR on mouse testis cDNA the whole transcript spanning from the AUG codon of LOC432534 to the stop codon of *Pms1*. We showed by RT-PCR that this new isoform also exists on testis cDNA from human, dog, cat and chicken. This new isoform 2 of *Pms1* displayed the same expressional post meiotic pattern than the isoform 1 (NM_028669) (data not shown).

**Figure 8:**
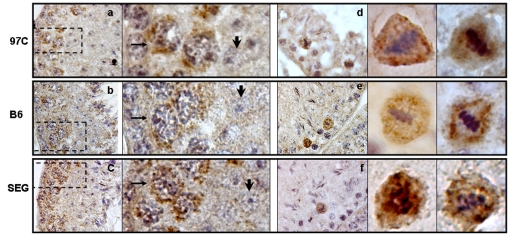
Immunohistochemistry using an anti-FIGNL1 antibody on mouse testes sections. From a cell type point of view, the location of the protein does not appear different between 97 C, B6 and SEG. (a and d) correspond to images from 97 C testes, (b and e) images from B6 testes and (c and f) images from SEG testes taken at objective ×100. (a, b and c) show stages VI–VIII of the spermatogenesis, close to spermiation. The Fignl1 is concentrated in the cytoplasm of mid-pachytene spermatocytes in a crescent-like pattern (see thin arrows in the enlarged views of the dashed area in a, b and c). To note the absence of staining in the spermatids (large arrows in the enlarged views). (d, e and f) show a stage XII with immunoreactive spermatocytes in metaphase. Notice in the enlargements presented at the right side of d, e and f pictures, the very particular location of Fignl1 around the achromatic spindle. This observation is representative of metaphases in B6 mice. In 97 C, a diffuse location of Fignl1 around the chromosomes could also be observed.

**Figure 9:**
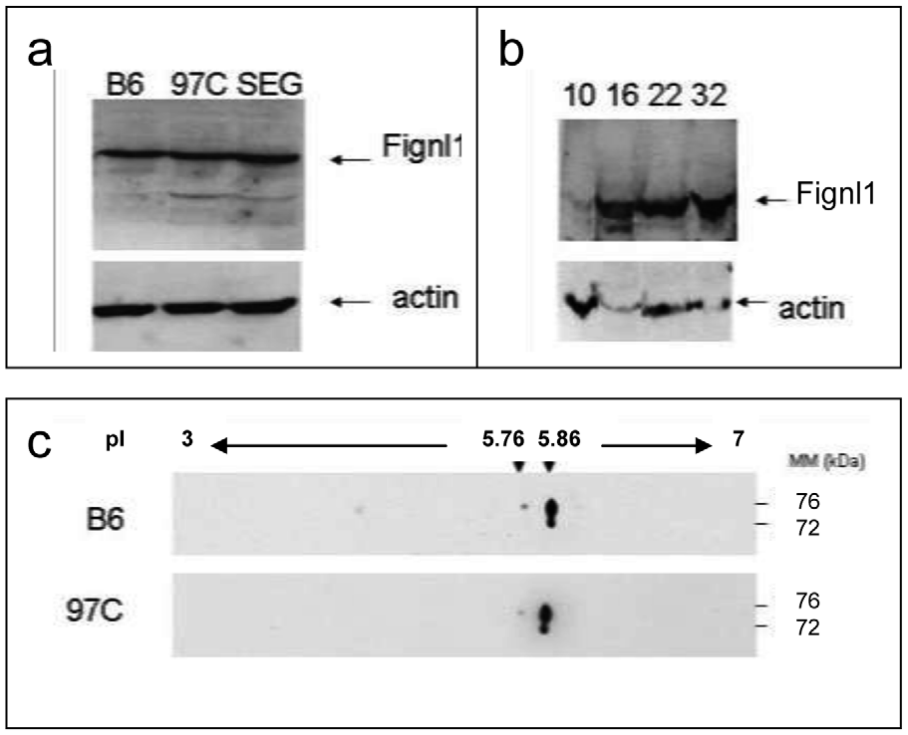
Biochemical characterization of Fignl1 from 97 C, B6 and SEG testes. (a) Western blot revealed by anti-FIGNL1 antibody on extracts of B6, 97 C and SEG testes (50 µg of protein extract per lane). β-actin is taken as an internal control of loading. The Fignl1 band migrates around 74 kDa. There is no obvious difference in size and quantity between the two mouse species, as well as in the 97 C IRCS. The alternate splicing (Figure 7) should generate a ∼6 kDa peptide that was not visible in our electrophoresis conditions. (b) Western blot revelation of the Fignl1 during the first wave of the spermatogenesis in B6 testis from 10 to 32 days post partum (DPP). Fignl1 is not detectable at 10 DPP, date of the meiosis entry but is observed at 16 DPP, and afterward, when spermatocytes appear in the tubules.). β-actin is taken as an internal control of loading. To note: the 7 kD isoform described in the text could not be detected in this 12% polyacrylamide gel. (c) Two-dimensional Western blot of protein extracts of B6 and 97 C testes revealed by anti FIGNL1. Fignl1 migrates as two principal spots at 76 kDa and 72 kDa for a same 5.86 pI. The 2D migration profile was the same for B6 and 97 C.

Contrarily to *Pms1*, *fidgetin like 1* (*Fignl1*) is reported to be expressed in pachytene spermatocytes and to a lesser extent in spermatogonia. *Fignl1* encodes a protein belonging to the group of ATPases Associated with diverse cellular Activities (AAA) [17] in the subgroup of meiotic proteins which also comprises *fidgetin*, *fidgetin-Like 2*, *katanin*, *spastin*, and *SKD1* genes [18]. We realized an immunolabeling of adult testis sections with an anti-FIGNL1 antibody. Histological analysis of B6, 97 C and SEG testis sections revealed a same localization of Fignl1 for the three strains, in spermatocyte cells (figure 8). A perinuclear staining was visible in the cytoplasm of pachytene spermatocytes (figures 8 a, b, c) and a strong immunoreactivity was also observed in metaphase spermatocytes (figure 8 d, e, f). By contrast no signal was found in spermatid cells (large arrows in enlarged figures 8 a, b, c). These experiments revealed a quite striking exclusion of labeling of the spindle area in meiotic metaphase in B6, while in 97 C, and to a lesser extent in SEG, some metaphasic spindle area appeared labeled with anti FIGNL1. In agreement with the testis transcriptome data showing a same expression level for B6 and 97 C *Fignl1* (Table 1), a Western blot of adult testes from B6, SEG and 97 C did not reveal differences in expression levels of Fignl1 protein detected at the 74 kDa expected size (Figure 9a). An analysis by WB of the presence of Fignl1 during the first spermatogenetic wave in B6 testis showed that it was still undetectable at 10 DPP, i.e. at a moment close to the entry in male meiosis. It was detected at 16 PDD and persisted afterward, in concordance with its expression at the spermatocyte stage (figure 9b).

To better characterize Fignl1, we performed a two-dimensional electrophoresis/WB of B6 and 97 C testis extract (Figure 9c). The profile of 2D-migration of Fignl1 according to the isoelectric point (pI) and the molecular mass (MM) was the same for 97 C and B6 testis, i.e. the protein migrated as two principal dots at 76 kDa and 72 kDa for a same 5.86 pI. A third faint but detectable spot migrated at 76 kDa and 5.76 pI. These values are slightly different from the theoretical values (74 kDa MM and 6.04 pI), signifying moderated posttranslational modifications. The pI values slightly more acidic compared to the theoretical value (difference of 0.18 and 0.28 unit) could correspond to two phosphorylated sites and one actelylated site of the Fignl1 protein as found by high throughput experiments [19], [20], [21]. In sum, Fignl1^spretus^ protein expressed in 97 C testis does not appear biochemically different from Fignl1^dom^. protein expressed in B6.

These overall results that describe for the first time the presence and the subcellular localization of Fignl1 in mammalian testis indicate that Fignl1 is mainly localized in meiotic germ cells, the spermatocytes, with a subtle anomaly of its localization in 97 C testis. Since spermatocytes are functionally impaired in 97 C testes, our results on Fignl1 together with the exclusion of all the other genes present in the minimal fragment strengthen the implication of Fignl1 in the *Ltw1* QTL.

In the literature, three studies give consensual insights on *Fignl1* function reporting action on proliferative/differentiation processes. Very importantly, the loss of *Figl1* function by injection of anti-*Figl1* siRNA in *C. elegans* [22] leads to the accumulation of mitotic nuclei inside the proliferative zone of the gonad, preventing the passage of the cells to meiosis. Another study showed that in transiently transfected mouse osteoblast cell model (MC3T3-E1) Fignl1 inhibits osteoblast proliferation and stimulates their differentiation [23]. The third study, using a silencing approach in proliferating MIN6 cells (derived from pancreatic β-cells), suggested that Fignl1 was implicated in adequate accomplishment of cell cycle by controlling cell survival [24]. Thus, if Fignl1 function is impaired in the 97 C testis, it could lead to a slowing down of germ cell differentiation with, in consequence, an increase of apoptotic cells (it is natural to think that defects in the normal progression of the meiotic cell cycle must to end with apoptosis to avoid formation of defective gametes). The extension of meiosis duration during the first spermatogenetic wave in 97 C, is strictly consistent with a role of Fignl1 in the regulation of meiosis. The observations reported in mouse osteoblasts, pancreatic cells and especially in *Caenorhabditis* gonads contribute to propose *Fignl1* as an important novel agent of control of meiosis dynamics. Since Fignl1 belongs to the AAA protein family whose function depends on ATP consumption, it is possible that proteins involved in energy supply to other proteins of musculus genomic origin, could malfunction on a spretus factor. This hypothesis does not exclude the degradation kinetics that we suggest as plausible for explaining Fignl1 spretus dysfunction in the musculus context.

In summary, all the data collected in the literature and those obtained by our own experiments converge to present *Fignl1* as a strong candidate for the testicular phenotype of 97 C mice. Indeed, compared to the 11 genes of the refined region, *Fignl1* is the only gene which is expressed in the cell type related to the phenotype, has a known role in a function (especially the passage from mitosis to meiosis in C. elegans) related to the phenotype and harbors several non synonymous SNPs differentiating the *spretus* alleles from the *musculus* alleles of *Fignl1*. The amount of Fignl1 could be important for regulating meiosis length, this amount may be affected either by an increased synthesis or by a protection against degradation. We did not observe a specific increase in the level of gene expression in 97 C testes; this, in accordance with C. elegans data [19] could lead to speculate that this degradation could be the crucial mechanism. As a hypothesis, it is conceivable for instance that ubiquitinylation systems of Mus musculus are less efficient to degrade Mus spretus versions of Fignl1, leading to an accumulation of the protein able to slow down the meiosis process. This slowing meosis will result in apoptosis or in an increased occurrence of sperm anomalies, thereby explaining the teratozoospermia observed in 97 C.

### 4) How the *spretus* fidgetin like1 allele could affect male meiosis in 97 C mice?

Since the expression level of *Fignl1* is the same in adult 97 C and B6 testis (see Table 1), we ruled out the hypothesis that its promoter/regulation region, from *spretus* origin, does not fit with transcription factor of B6 origin, that should act on this promoter. Rather, the problem takes its origin from a Fignl1 *^spretus^* dysfunction in the B6 context, caused by evolutionary divergences in the sequence. We found nine non-synonymous polymorphisms between *Fignl1^dom^* and *Fignl1 ^spretus^* alleles. Eight out of the 9 mutations were found in the N terminal sequence, affecting neither the AAA nor the Vsp4 domains of Fignl1 (figure 7). In a review of 2007, White and Lauring put forward the divergent N-terminal domain utilized by these AAA proteins to interact with adaptor partner proteins [25]. So, it is possible that Fignl1 and its protein partners have co-evolved differently over the 2MYr separating *Mus spretus* and *Mus musculus* species, preventing an efficient interaction between Fignl1 *^spretus^* allelic form and its *Mus musculus* protein partners. To note, a sequence alignment of *spretus* Fignl1 with the sequence of other vertebrate species showed that among the nine amino acid substitutions, H379Y and M397K, touched conserved sequences and was specific to *spretus* Fignl1 (Figure S3).

Another difference between *spretus* and *musculus* Fignl1 was the expression by 97 C and SEG mouse strain testes of a truncated *Fignl1* isoform in addition to the full length isoform which is present in B6 (figure 7b). This truncated isoform corresponds to an alternative splicing between acceptor and donor AG site (position: 401 bp and 1706 bp one NM_021891.3 transcript) removing approximately 1400 bp of the coding region. Interestingly, these cryptic splicing sites do exist in the B6 isoform, but seem to be discarded by the splicing machinery, which suggests that some of the synonymous polymorphisms present in the *spretus* isoform either affect or create an exonic splicing silencer element. This truncated isoform might encode a short peptide corresponding to the 60 first amino acids of Fignl1. An *in silico* analysis of this short polypeptide on ELM (Eukaryotic Linear Motif resource) functional prediction site (http://elm.eu.org/) showed that this peptide contains several putative functional signals, and notably a destruction motif, targeted by the anaphase-promoting ubiquitin ligase complex APC/C. The targets of the APC/C are degraded to ensure the correct progression in the cell cycle through mitosis and meiosis [26]. This short polypeptide may act as a competitor of the full length protein in this degradation process. Interestingly, it has been shown that, in *C. elegans*, Figl1 is targeted specifically by the CUL-3/MEL-26/E3 ligase complex resulting in its degradation during meiotic stages [22]. The authors suggest that this degradation might be necessary to ensure the mitotic to meiotic passage of the worm germ cells. Thus a defect in the degradation of Fignl1 during meiosis could lead to a lengthening of the process. Interestingly, this short isoform, when fused with eGFP and transfected in cultured cells, displays a cytoplasmic expression, in a dotted pattern, different from that of eGFP alone, arguing in favor of existence of degradation signal in the N terminus domain of Fignl1 protein (data not shown).

In this study, we have carried out the positional cloning of a gene able to modulate testis weight by interfering with meiosis. For achieving this, we used a mouse model where *Mus musculus* genome is locally substituted by *Mus spretus* genome. The identification of Fignl1 as responsible for the phenotype has been obtained by narrowing down the minimal interval containing the gene on chromosome 11, by eliminating the other genes present in the interval, according to experimental data and bioinformatics and by establishing a parallel with the phenotype described in knocking down the homologous gene (*Figl1*) in *Cenorhabditis elegans*. It is clear that the generation of null mice or even better, a knock-in experiment substituting the spretus *Fignl1* in a B6 genomic context, would directly prove the involvement of the gene. However, our study clearly suggests that Fignl1 has an important role in controlling male meiosis in mice. Dissection of molecular events leading to meiotic impairment in our original model would contribute clarifying this complex spermatocyte prophase I regulation. This model should make it possible to find the molecular bases of the epistatic breakdown that we observe (i.e. meiotic impairment in 97 C but not in SEG or B6 strain), and to discover *Fignl1* genetically interacting genes. Eventually *Fignl1* deregulation and mutation should be investigated in infertile human male presenting with idiopathic meiotic arrest.

## Methods

### Ethics statement

The experimental procedures were conducted in accordance with the policies of the University and the Pasteur Institute (Paris) and the Guidelines for Biomedical Research Involving Animals. The animals were kept under standard conditions according to the recommendations for the use of animals in experimental designs and according to the “3R” rules. The work was performed under the agreement N/Ref RL-0801432-30801038, authorization 75–1463 obtained from the “Direction Départementale des services vétérinaires de Paris”.

### Animal housing

Parental strains, C57BL/6J (B6) and SEG/Pas, and 97 C IRCS were provided after weaning by The Pasteur Institute (Paris). IRCS were constructed according to a scheme previously reported [8], [10]. Animals were housed at the animal facility of the Cochin Institute, under normal conditions of light/dark cycle, temperature and free access to mouse food and water.

### Microsatellite genotyping

DNA was extracted from mouse tail fragments according to a classical procedure [27]. Three microsatellites on MMU6 (D6MIT224, D6MIT321, D6MIT313) and 12 microsatellites on MMU11 (D11MIT304, D11MIT72, D11MIT74 D11MIT129, D11MIT62, D11MIT259, D11MIT204, D11MIT150, D11MIT133, D11MIT148, D11MIT63 and D11MIT162) were used in order to precise the boundaries of the *spretus* segment in 97 C mouse strain genome and to genotype congenic sub-strains. Primer microsatellites were retrieved from the Mouse Genetic Informatic website of the Jackson Laboratory. PCR was performed using *Taq* DNA Polymerase (New England Biolabs). PCR products were loaded in a 2% nusieve, 5% agarose gel (Cambrex Bio Science Rockland, Inc).

### Generation of sub-congenic mice

97 C mice were crossed with B6 individuals in order to obtain heterozygous mice for the *spretus* fragments. A first F2 population was established and genotyped in order to separate the two 97 C *spretus* fragments located on MMU6 and MMU11. Individuals of both sexes harboring only one of these genomic fragments were selected and crossed in order to establish two congenic sub-strains, 97Crc6 and 97Crc11 respectively. Concerning 97Crc11 sub-strains, a second F2 population was established from a cross between 97Crc11 and B6 mouse then genotyped in order to find recombinations inside the spretus fragment. Such recombinant individuals were selected and crossed with B6 mice in order to obtain recombinants of both sexes which were crossed between them to get homozygous recombinant sub-strains.

### Whole Testis RNA extraction and cDNA synthesis by reverse-transcription

Total testis RNA from several SEG/Pas and 97 C males was extracted using TRIzol Reagent (Invitrogen, Carlsbad, CA, USA) according to the manufacturer's instructions. RNA extractions from the two testes of six males of each strain were pooled before DNase I treatment (Invitrogen, Carlsbad, CA, USA).

Testis RNA was reverse transcribed to obtain cDNA using either M-MLV Reverse Transcriptase (Invitrogen, Carlsbad, CA, USA) or Roche *Transcriptor* Reverse Transcriptase following manufacturer's protocols.

### *Mus spretus* allelic variant ORFs sequencing

PCR primers (primer sequences available upon request from the authors) were designed, based upon the B6 sequences in 5′ and 3′ UTR sequences surrounding the ORFs of *Grb10*, *Zpbp*, *Pms1Rik* and *Fignl1* genes. These primers were then used to amplify *Mus spretus* gene versions from testis cDNA extracted from SEG/Pas and 97 C males. PCR products were purified and sequenced. *Mus spretus* sequences were BLASTed against the B6 sequences, taken as the reference.

### Morphological and histological analysis of the reproductive organs

The male adult mice (8–9 week old) were killed by cervical dislocation and reproductive organs (testes, epididymis and seminal vesicles) were dissected and weighted. For the study of post-natal testis development and first wave of spermatogenesis, juvenile mice were killed by decapitation at day post partum (DPP) 9, 14, 21, 24, 28, 35, 42 and 49 and their testes were recovered and weighted. The adult and juvenile organs were either fixed for histological analysis or deep frozen at −80°C for further protein analysis.

For histological analysis, organs were immersed in DF2 fixative (35% absolute alcohol, 10% acetic acid, 2% formaldehyde, in distilled water) for 24 hr at room temperature then embedded in paraffin. Organs were sectioned (4 µm) then deparaffinized and stained by hematoxylin for histological tubule examinations.

### Immunohistochemistry

Testis sections were deparaffinized, rehydrated and then treated either by 0.2% TX-100 in PBS for 5 minutes or by 3×5 minutes microwave in citrate buffer (0.1 M sodium citrate and 0.1 M citric acid, pH 6.0) just before blockade of peroxidase activity by incubation with 3% H_2_O_2_ for 10 min. After non specific binding site saturation, sections were incubated with anti FIGNL1 antibody (Novus Biologicals) (1/100 or 1/50 in blocking solution) for one hour at room temperature and then with horseradish peroxidase-conjugated anti-rabbit antibody. The sections were finally treated with diaminobenzidine in the dark, washed and then rapidly counterstained with Hemalun de Mayer (RAL) and mounted in Eukitt medium (Labonord). Negative controls were performed by incubating sections with rabbit IgG (1/100) in place of the primary antibody.

### Protein extract and Western blot analysis

Protein from adult or juvenile testes were extracted by homogenization of whole testis in Laemmli lysis buffer with β-mercaptoethanol and protein extract were boiled 10 minutes at 95°C before loading on SDS-PAGE 12%gels.

For 2D separation, testis proteins were solubilized in 9 M urea, 4% CHAPS, 100 mM DTT and 2% IPG buffer at room temperature. Extracted proteins were separated in precast 3–10 IPG strips (GE Healthcare) and then in SDS-PAGE 12% gels for the second dimension.

The proteins were electro-transferred to a polyvinylidene difluoride membrane and then probed with rabbit anti-FIGNL1 antibody (1/1000) and HRP labelled anti rabbit IgG. HRP activity was detected using the ECL kit (GE Healthcare) and exposed to Kodak X-OMAT film (Fisher-Scientific)

### TUNEL assay

Sections of testes from adult mice were subjected to TUNEL assay. Fixed sections were deparaffinized, rehydrated and demasked by 20 µg/ml proteinase K (RNA grade, Invitrogen Corporation). Apoptotic cells were revealed using in Situ Cell Death detection kit, AP (Roche Diagnostics) that labels the DNA strand breaks with fluorescein-dUTP. Sections were counterstained with DAPI before two independent fluorescence microscopy examinations. Two hundred twelve and 161 tubule sections were examined for 97 C and B6 mice, respectively. Fluorescent cells (TUNEL positive) were counted in each tubule section on different fields observed at objective ×20.

### Statistical tests

The Student T test and the non parametric Kruskall and Wallis test were used to compare mean values and Chi square test for percentage value. Values were considered significant under the threshold of 0.05 (p<0.05).

## Supporting Information

Figure S1 Histological sections of epididymal duct at 28 and 35 days post partum in 97 C and B6 mice. At 35 DPP, we observed the presence of spermatozoa in the epididymal duct lumen of B6 mice whereas only abnormal/apoptotic round cells are visible in 97 C epididymis. These cellular elements are normally present in the epididymal duct lumen at 28 DPP consequent to the setting up of the first wave of spermatogenesis.
(PDF)

Figure S2 Expressional data of *Pms1* during the first wave of spermatogenesis. RT-PCR amplification of *Pms1* ORF from testis cDNA of B6 mice at 10, 16, 20, 22, 24 and 30 days post partum. The specific amplification product is undetectable at 10, 16, and 20 DPP when germ cells are only represented by spermatogonies and spermatocytes in the tubules. It became observable from 22 DPP, concomitantly with the apparition of spermatids which begun to differentiate in the tubules.
(PDF)

Figure S3 Phylogenetic conservation of Fignl1 across vertebrate species. In blue are represented amino-acids of the B6 type, while in yellow are represented amino-acids of the spretus type. The amino-acids located at positions 379, 397 and 599 are apparently strictly specific of spretus SEG/Pas. It is interesting to notice that S99C is generally specific of non mammal species (except the monotremata).
(PDF)
