# Supplementary figures and images for: Fidgetin-Like1 Is a Strong Candidate for a Dynamic Impairment of Male Meiosis Leading to Reduced Testis Weight in Mice
Source: PLoS One. 2011 Nov 16;6(11):e27582. doi: 10.1371/journal.pone.0027582 (PMC3217987; doi:10.1371/journal.pone.0027582)

10 16 20 22 24 30 PND

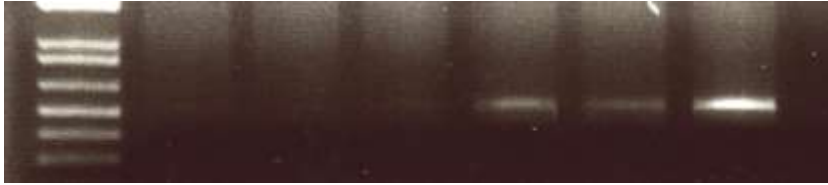

Northern blot

*4930415F15Rik*

Supplement: Figure S1 — Histological sections of epididymal duct at 28 and 35 days post partum in 97 C and B6 mice. At 35 DPP, we observed the presence of spermatozoa in the epididymal duct lumen of B6 mice whereas only abnormal/apoptotic round cells are visible in 97 C epididymis. These cellular elements are normally present in the epididymal duct lumen at 28 DPP consequent to the setting up of the first wave of spermatogenesis. (PDF) [file pone.0027582.s001.pdf]

10 16 20 22 24 30 PND

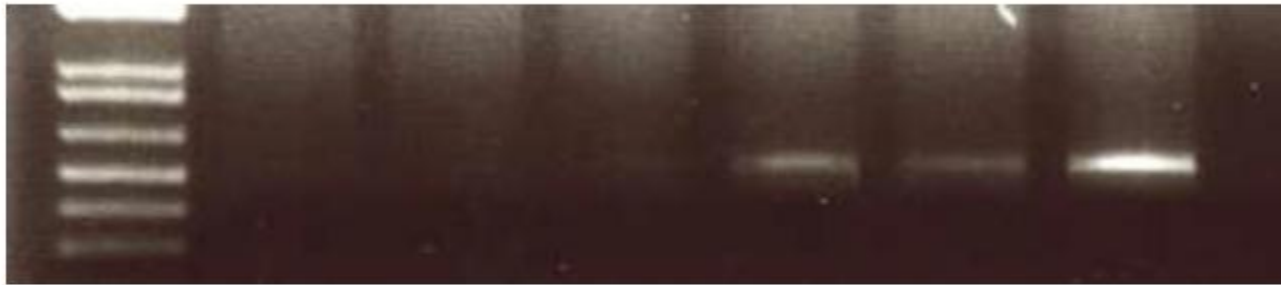

qRT-PCR

*4930415F15Rik*

Supplement: Figure S2 — Expressional data of Pms1 during the first wave of spermatogenesis. RT-PCR amplification of Pms1 ORF from testis cDNA of B6 mice at 10, 16, 20, 22, 24 and 30 days post partum. The specific amplification product is undetectable at 10, 16, and 20 DPP when germ cells are only represented by spermatogonies and spermatocytes in the tubules. It became observable from 22 DPP, concomitantly with the apparition of spermatids which begun to differentiate in the tubules. (PDF) [file pone.0027582.s002.pdf]
