# Supplementary material for: Fidgetin-Like1 Is a Strong Candidate for a Dynamic Impairment of Male Meiosis Leading to Reduced Testis Weight in Mice
Source: PLoS One. 2011 Nov 16;6(11):e27582. doi: 10.1371/journal.pone.0027582 (PMC3217987; doi:10.1371/journal.pone.0027582)
Supplement: Figure S3 — Phylogenetic conservation of Fignl1 across vertebrate species. In blue are represented amino-acids of the B6 type, while in yellow are represented amino-acids of the spretus type. The amino-acids located at positions 379, 397 and 599 are apparently strictly specific of spretus SEG/Pas. It is interesting to notice that S99C is generally specific of non mammal species (except the monotremata). (PDF) [file pone.0027582.s003.pdf]

Position AA	99	121	152	172	174	222	379	397	599
Homo-sapiens	S	S	V	N	A	K	H	M	E
Pan-troglodytes	S	S	V	N	A	K	H	M	E
Pongo-abelii	S	S	V	N	A	K	H	T	E
Macaca-mulatta	S	S	V	N	A	K	H	M	E
Callithrix-jacchus	S	S	V	N	A	K	H	M	E
Equus-caballus	S	N	V	K	A	K	H	M	E
Bos- taurus	S	S	V	N	A	K	H	M	E
Canis-familiaris	S	N	V	N	A	K	H	M	E
Ailuropoda-melanoleuca	S	N	V	N	A	K	H	M	E
Mus-musculus	S	C	A	S	G	A	H	M	D
Mus-spretus	C	S	V	G	V	T	Y	K	N
Rattus-norvegicus	S	T	I	S	V	T	H	M	D
Oryctolagus-cuniculus	S	S	V	N	V	R	Q	M	E
Gallus-gallus	C	C	A	G	S	G	F	M	E
Taeniopygia-guttata	C	C	A	G	A	G	L	M	E
Ornithorhynchus-anatinus	C	R	V		G	S	P	T	Q
Xenopus-laevis	C	S	I	A	P	I	S	M	E
Xenopus-tropicalis	C	S	I	T	P	I	A	M	E
Danio-rerio	N	C		L	Q	G	Q	M	V

Supplementary figure 3
L'Hôte et al
